# Current Progress in CAR-T Cell Therapy for Solid Tumors

**DOI:** 10.7150/ijbs.34213

**Published:** 2019-09-07

**Authors:** Shuo Ma, Xinchun Li, Xinyue Wang, Liang Cheng, Zhong Li, Changzheng Zhang, Zhenlong Ye, Qijun Qian

**Affiliations:** 1 Shanghai Baize Medical Laboratory, Shanghai, China; 2 Department of Pathology and Laboratory Medicine, Indiana University School of Medicine, Indianapolis, Indiana, USA; 3 Shanghai Cell Therapy Research Institute, Shanghai, China; 4 Shanghai Engineering Research Center for Cell Therapy, Shanghai, China

**Keywords:** CAR-T cells, chimeric antigen receptor, solid tumors, companion diagnostics, CTC

## Abstract

Cancer immunotherapy by chimeric antigen receptor-modified T (CAR-T) cells has shown exhilarative clinical efficacy for hematological malignancies. Recently two CAR-T cell based therapeutics, Kymriah (Tisagenlecleucel) and Yescarta (Axicabtagene ciloleucel) approved by US FDA (US Food and Drug Administration) are now used for treatment of B cell acute lymphoblastic leukemia (B-ALL) and diffuse large B-cell lymphoma (DLBCL) respectively in the US. Despite the progresses made in treating hematological malignancies, challenges still remain for use of CAR-T cell therapy to treat solid tumors. In this landscape, most studies have primarily focused on improving CAR-T cells and overcoming the unfavorable effects of tumor microenvironment on solid tumors. To further understand the current status and trend for developing CAR-T cell based therapies for various solid tumors, this review emphasizes on CAR-T techniques, current obstacles, and strategies for application, as well as necessary companion diagnostics for treatment of solid tumors with CAR-T cells.

## Introduction

Immunotherapy with CAR-T cells has achieved tremendous successes in treatment of hematological malignancies. Two CD19-targeting CAR-T cell products, Kymriah from the Novartis (East Hanover, NJ USA) and Yescarta from the Kite Pharma (Santa Monica, CA USA), have been approved by the US FDA for treating B cell acute lymphoblastic leukemia (B-ALL) and diffuse large B-cell lymphoma (DLBCL), respectively 1. However, due to intricacies of solid tumors and their locations in the human body, treatment of solid tumors with CAR-T cells is facing multiple obstacles, such as the hostile tumor microenvironment, on-tumor/off-tumor toxicities, and undesired antigen specificity 2. Many strategies and approaches have been tried to overcome these obstacles, including arming CAR-T cells with knock-out of PD-1 expression or secretion of cytokines/chemokines and using CAR-T cells in combination with other treatments 3-5. Despite these efforts, there are still no CAR-T cells clinically approved for solid tumor treatment so far. Encouragingly and optimistically, in this landscape, more than forty clinical trials in treatment of solid tumors by CAR-T cells have been registered in China alone (Table 1) 6.

Beside the focuses on the aspects of treatment, companion diagnostics are increasingly recognized as playing important roles in patient screening, treatment regimen, efficacy evaluation, and real-time monitoring of CAR-T cell therapies. Therefore, in this review, we focus on current CAR-T techniques**,** obstacles, strategies for overcoming these obstacles, as well as necessary companion diagnostics in treatment of solid tumors with CAR-T cells.

## Overview of CAR-T techniques

Chimeric antigen receptor (CAR) is the core component of CAR-T, which endows T cells with the ability to recognize tumor antigens in a HLA-independent manner and enables them to recognize more extensive target antigens than natural T cell surface receptor (TCR) 7. A basic CAR includes a tumor-associated antigen (TAA) binding domain (usually from the scFv fragment of antigen-binding region of the monoclonal antibody), an extracellular hinge domain, a transmembrane domain and an intracellular signal domain 7, 8.

The activation of T cells mediated by the first generation of CAR is accomplished by the tyrosine activation motif on CD3ζ chain or FcεRIγ 9. CD3ζ chain can provide “signal I” for T cell activation, cytolysis, regulation of IL-2 secretion and anti-tumor activity in vivo 10, 11. However, the anti-tumor activity of the first generation of CAR modified T cells is limited in vivo, and the decreased proliferation of T cells ultimately leads to apoptosis 12, 13. The second generation of CAR adds a new costimulatory signal in the intracellular region, which enlarges the original “signal I” derived from TCR/CD3 complex 14, 15. Many studies have shown that compared with the first generation of CAR, the second generation of CAR carrying “signal II” has the same antigen specificity, increased T cell proliferation and cytokine secretion, enhanced secretion of anti-apoptotic proteins, and delayed cell death. The ubiquitously used costimulatory molecule is CD28 16, 17, which have been gradually been replaced with CD137 (4-1BB) 18. In addition, an idea of using NK cell receptor CD244 has also been proposed to promote sustained activation and proliferation of CAR-T cells 19.

In order to further improve the design of CAR, many studies began to focus on the development of the third generation of CAR, including not only “signal I”, “signal II”, but also additional costimulatory signals. Studies using different targets and costimulatory signals were conducted to compare the results of the second and third generation of CARs and obtained quite encouraging experimental results 20, 21. Zhong et al. combined CD28 and 4-1BB costimulatory signaling domains to construct a CAR specific for prostate-specific membrane antigen (PSMA). This group demonstrated that the CAR they constructed was able to induce strongest PI3K/Akt activation and Bcl-X_L_ expression in vitro, and the least apoptosis in transduced peripheral blood CD8^+^ T cells 20. Targeting a different tumor marker, MUC1, Wilkie et al. designed a CAR containing a fused CD28/OX40/CD3ζ endodomain. Using the engineered CAT-T cells and upon MUC1 stimulation, they showed that these CAR-T cells were able to secrete proinflammatory cytokines indicative of both type-1 (IFN-γ) and Th17 (IL-17) differentiation in vitro. It was noteworthy that IL-17 has been known as tissue destructive cytokine in autoimmune disease animal models, although its anti-tumor effect is still to be elucidated 20, 21.

Up to date, it is still uncertain which design is better between the second generation and the third generation of CAR. Additionally, the second and third generations of CARs have their own on-going clinical trials in the US, China and Europe, and the development and outcome of these clinical trials are being closely watched.

## Obstacles in treating solid tumors with CAR-T cells

### 1. CAR-T cells traffick to the tumor sites

To bind to their target proteins on the surface of tumors, CAR-T cells first need to traffick to tumor sites. This is the fundamental prerequisite for T cell immunotherapy to work properly. Unlike the hematological malignancies, in the case of solid tumors, T cells trafficking to and infiltrating into tumor sites are oftentimes greatly limited by the immunosuppresive microenvironment 22. Also unlike hematological malignancies that are easy to be targeted and reached by CAR-T cells, some of chemokines such as CXCL1 23, CXCL12, and CXCL5 24, 25 secreted by solid tumors prevent T cells from trafficking toward and infiltrating into the tumor lesions. Due to lack of corresponding chemokine receptors expressed on T cells, it is difficult for them to traffic and infiltrate into tumor sites, drastically impeding the ability of CAR-T cells for their designed immuno-cytotoxicity for killing tumor cells. Therefore, in order to overcome this obstacle, T cell will need to be modified to express chemokine receptor that matches the corresponding tumor-derived chemokine. An early study conducted by Kershaw et al. demonstrated that chemokine receptor (CXCL1 receptor)-engineered T cells can greatly drive themselves to migrate toward melanoma cells 23**.** This study demonstrated the feasibility that T cell traffic can be re-directed to tumor sites by chemokines secreted by tumor cells.

### 2. CAR-T cells infiltrate into tumors

Once CAR-T cells successfully traffic to tumor sites, infiltrating into tumor microenvironment is a required critical step to exert anti-tumor effects. This is a very highly dynamic and regulated complex step: it involves rolling, adhesion, extravasation and chemotaxis 26. Solid tumors have unique histopathological features, such as concentrated blood vessels 27, as well as tumor-associated fibroblasts and myeloid cells forming extracellular matrix (ECM). While these features favor the growth of solid tumors, they impose the difficulty for T cell infiltration in tumor sites 27, thus preventing the continuous contact between T cells and tumor cells that is necessary for T cells to exert cytotoxic antitumor effects.

### 3. The immunosuppressive state of microenvironment in solid tumors

The immunosuppressive microenvironment within solid tumor has special histopathological features as manifested by high density blood vessels, extensive vascular leakage, poor integrity of tissue structure, and others 2. These changes result in hypoxia, low pH, immune suppressor cells, augment of inhibitory checkpoint, and more tumor-derived cytokines 28-30. Following are three major factors that affect the antitumor effects of T cells:

#### 3.1 Immune suppressor cells

Solid tumors generally consist of a large number of immune suppressor cells, such as regulatory T cells (Tregs), myeloid-derived suppressor cells (MDSCs), and tumor-associated macrophages. These cells all play roles to protect solid tumor cells from being killed by the host's immune system. Theoretically, costimulatory molecules, such as CD28 and CD137, may benefit CAR-T cell activation and survival in tumor sites 31, 32. In addition, blocking granulocyte-macrophage colony stimulating factor (GM-CSF) dependent MDSC expansion and PD-L1 expression on MDSC may be a potential approach to enhance the antitumor effects of CAR-T cells 33.

#### 3.2 Tumor-derived cytokines

Tumor-derived cytokines are soluble factors that deter the efficacy of cancer immunotherapy for solid tumors. Transforming growth factor-β (TGF-β) is an inhibitory tumor cytokine that plays a major role in alleviating the antitumor response. TGF-β downregulates CD8^+^ effector T cell function and upregulates Treg maturation 27. In contrast, neutralizing TGF-β by antibody or small molecular drugs improves CD8^+^ T cell functions and enhances the antitumor response by increasing the number, infiltration and persistence of adoptively transferred T cells. Moreover, the activation of cytokines, such as interleukin (IL)-2 and IL-15, improves the antitumor effects of CAR-T cells 34. IL-12, in particular, alters the tumor microenvironment 35, eliminates antigen-negative tumor cells 36, and prolongs the survival of T cells to increase efficacy of immunotherapy through recruiting and activating macrophages and other innate immune cells 27. In addition, to alter the tumor microenvironment, armored CAR-T cells have been generated to secrete proinflammatory cytokines to improve CAR-T cell efficacy within the tumor microenvironment 37.

#### 3.3 Checkpoint inhibitory ligands

Inhibitory immune-checkpoint ligands induce the suppressive function of the immune response and are usually overexpressed in solid tumors. For example, PD-L1 is ligand for PD-1 receptor and inhibits CAR-T cell activation by binding to PD-1. PD-L1 has the two function motifs, immune-receptor tyrosine-based inhibitor motif (ITIM) and immune-receptor tyrosine-based switch motif (ITSM), which are involved in dephosphorylation of TCR-associated CD3 and zeta chain-associated protein kinase 70 (ZAP70) 27, 38. Blocking PD-1 through specific antibody, shRNA, or dominant negative receptor restored the function of Mesothelin-targeting CD28 CAR T cells, and CAR-T cells with constitutive anti-PD-1 secretion were more functional, expandable, and efficient in tumor eradication than parental CAR-T cells in a human lung carcinoma xenograft mouse model 39, 40. Checkpoint blockades are being applied to many clinical trials in combination treatment with CAR-T cells 41, 42.

### 4. The immune-related adverse events(irAEs)

One of the major challenges in CAR-T cell therapy for solid tumors is the irAEs 43-46. In 2010, death of a patient with colon cancer metastasis to the lung and liver following ERBB2-targeting CAR-T cell therapy was reported. The cause may be due to the recognition of CAR-T cells to low levels of ERBB2 on lung epithelial cells and resulted in triggering the release of remarkable amount of cytokines 43. There are two major mechanisms leading to the increased toxicity observed with CAR-T cell therapy:

#### 4.1 On-target/on-tumor toxicity

The most life-threatening toxicity following CAR-T cell immunotherapy is on-target/on-tumor toxicity, which relates to adverse effects, such as cytokine release syndrome (CRS). Upon binding to antigens on target tumor cells, infused CAR-T cells are extensively activated, leading to the release of a large number of inflammatory cytokines. The CRS symptoms include fever, fatigue, nausea, vomiting, diarrhea, rashes, delirium, hallucinations, hypotension, and even severe multiple organ failure 44, 47. The CRS has been classified into five grades according to the Common Terminology Criteria for Adverse Events (CTCAE) ranging from a mild reaction/grade 1 to death/grade 5. The level of the CRS severity is believed to be strongly correlated with tumor burden. IL-6 appears to be a major mediator of the CRS 48, and thus the key strategy to ameliorate this side effect is to block IL-6 directly with the IL-6R inhibitor tocilizumab 49. In addition to tocilizumab, new cytokine-directed approaches might be considered to overcome the CRS.

#### 4.2 On-target/off-tumor toxicity

Another type of toxicity originates from binding of CAR-T cells to target antigen that is also expressed on normal cells. This toxicity may lead to destruction of healthy cells and organs 43. In the treatment of neuroblastoma, the fatal neurotoxicity was observed in high-affinity GD2-specific CAR-T cell therapy, suggesting that GD2 may be an inappropriate targeting antigen for CAR-T cell therapy 50. Most of the CAR-T cell target antigens seem not to be tumor specific and shared by normal cells. Therefore, antigen specificity becomes a crucial factor for CAR-T therapy. To reduce the risk of this toxicity, selectivity of a safer antigen for CAR-T cells is critical and may be improved by utilizing dual CAR targeting, and modulating the sensitivity of single-chain variable fragment (scFv) 51. Toxicity can also be minimized by controlling CAR-T cell activity through manipulating CAR expression and introducing a switch that can be triggered in the severe condition of the irAE 52, 53.

### 5. Antigen specificity

Tumor cells exhibit different morphologic and phenotypic profiles. One of the predominant biological characteristics of solid tumors is their heterogeneity. Tumor heterogeneity significantly affects immunotherapy efficacy due to the immune target may limit to a certain population of solid tumor cells. Therefore, to improve target specificity and eliminate toxicity are necessary in CAR-T cell therapy. EGFR variant III (EGFRvIII) is believed to be the only tumor-specific antigen (TSA) for CAR-T cells. It is found to be exclusively expressed on human cancer cells rather than normal healthy cells 54-56, EGFRvIII-targeting CAR-T cells precisely target tumor cells and are helpful to increase efficacy and reduce toxicity.

The shortage of TSAs severely limits the use of CAR-T therapy. Therefore, targeting tumor-associated antigens (TAAs) is an alternative method to overcome the shortage of TSAs. Most of ongoing clinical trials of CAR-T cell therapies for solid tumors are using these TAAs 57. Attention should be paid to use of this CAR-T strategy because it may cause damage of normal tissues as TAAs are not only overexpressed on tumor cells but also on some normal cells.

### 6. Strategies to refine CAR-T cells for treatment of solid tumors

To overcome the obstacles mentioned above, we need to improve T cell efficacy and adopt new strategies that are discussed as below:

#### 6.1 Improving CAR-T cell trafficking and infiltration

**Figure 1:**
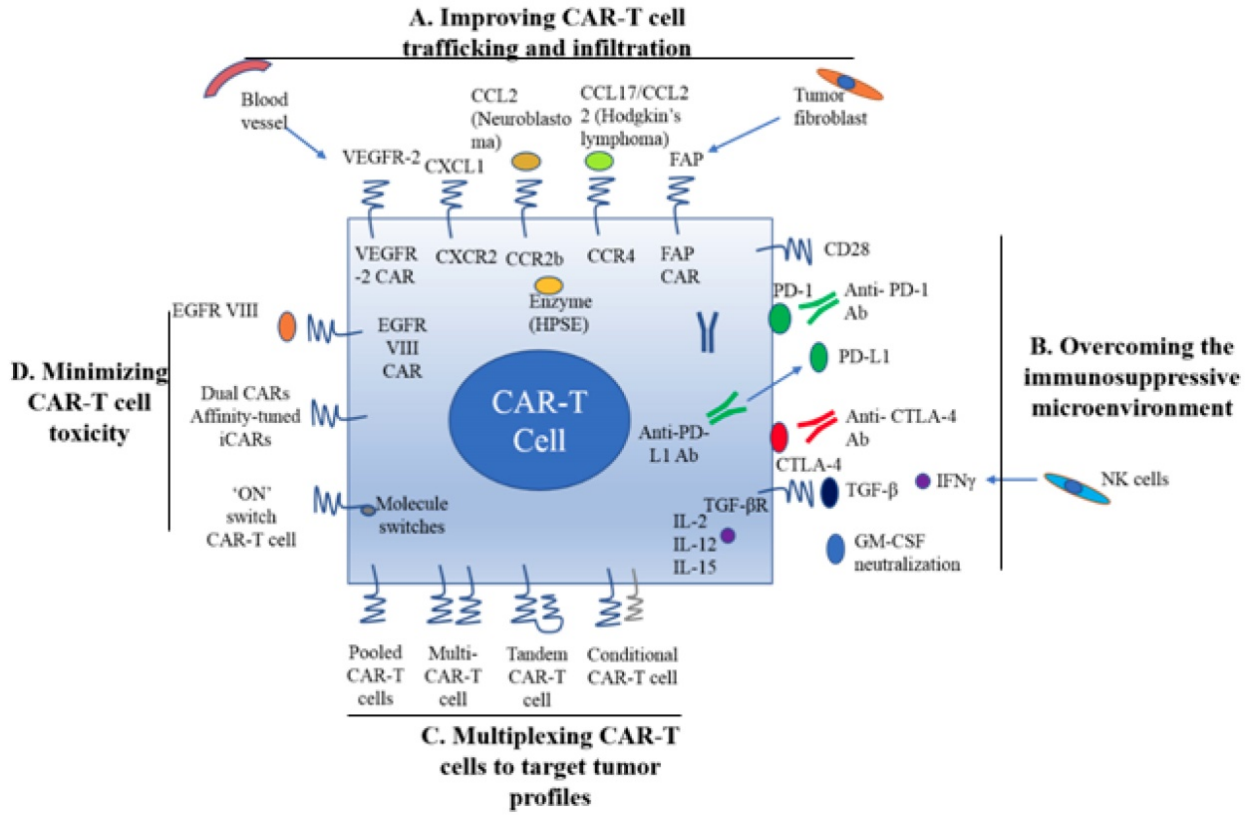
** A. Improving CAR-T cell trafficking and infiltration.** 1) Targeting the tumor stroma or vasculature: tumor fibroblasts are depleted by FAP-targeting CAR-T cells to inhibit tumor growth. 2) ECM consumption by a secreted enzyme: the ECM (heparan sulfate proteoglycans) can be disintegrated by HPSE. 3) Homing to tumors expressing chemokine receptors (CCR4, CCR2b): genetically modified CAR-T cells express chemokine receptor(s) matching the tumor chemokine to facilitate migration to the tumor cells. **B. Overcoming the immunosuppressive tumor microenvironment.** 1) Anti-PD-L1-secreting CAR-T cells: human NK cells are recruited to the tumor site through secretion of anti-PD-L1 antibodies from CAR-T cells. 2) CAR-T cell therapy is potently enhanced by PD-1 blockade. 3) Blockade with the IL-10/TGFβ receptor. 4) Proinflammatory cytokines secreted by armored-CARs and TRUCKS (IL-12 showed an increased antitumor efficacy). **C. Multiplex CAR-T cells to target the tumor profile.** 1) Pooled CAR-T cells: multiple single-targeting CAR-T cells are mixed together. 2) Multi-specific CAR-T cells: one bispecific CAR-T cell consists of two specific CARs. 3) Tandem CAR-T cells: two different CARs connected in tandem possessing a common intracellular domain. 4) Conditional CAR-T cells: one CAR has a CD3ζ signaling domain, and the other has a costimulatory domain. **D. Minimizing CAR-T cell toxicity.** 1) EGFRvIII CAR: EGFRvIII is the only truly tumor-specific antigen that is completely restricted to human cancer, such as glioblastoma. 2) Dual CAR: the first CAR activates T cell function through the CD3 signaling domain, and the second CAR contains CD28/CD137 to co-stimulate the signaling function. 3) Affinity-tuned CAR. 4) Inhibitory CAR (iCAR): normal cells are maintained safely because of negative signaling by iCARs that only have antigens that are expressed on normal cells. 5) 'ON' switch CAR-T cells: a CAR molecule is attached with a costimulatory CD3ζ signaling domain that can only be activated in the presence of a small molecule acting as an 'ON' switch. **FAP:** fibroblast activation protein; **ECM:** extracellular matrix; **HPSE:** heparanase; **TRUCKs:** T cells redirected for universal cytokine killing; **CAR:** chimeric antigen receptor. Note: The lines represent what A, B, C and D include respectively.

As mentioned earlier, CAR-T cell trafficking requires the establishment of chemotaxis migration between chemokines secreted by tumor cells and chemokine receptors on effector T cells. Different tumor types produce various chemokines so that a corresponding chemokine with its appropriate chemokine receptor is a critical factor for successful trafficking of T cells to tumor sites 22. T cells engineered with the chemokine receptor CXCR2, which binding to the ligand CXCL1 on tumor cells, have been demonstrated to effectively traffick toward melanoma (Figure 1A) 23. Di Stasi et al. demonstrated that the anti-lymphoma effects of T cells were improved by co-expression of CCR4. CCR4 can enhance the homing of CAR-CD30-modified T cells to CD30^+^ Hodgkin lymphoma cells by its secreted CCL17 (ligand for CCR4) 58. Similarly, the antitumor effects towards malignant pleural mesothelioma and neuroblastoma were improved by CCR2b expression of mesothelin- and GD2-targeting CAR-T cells, respectively 59. The local delivery of IL-7 and CCL19 by CAR-T cells improved immune cell infiltration and CAR-T cell survival in the tumor 60. Conversely, tumor cells mediate the secretion of chemokines from suppressive immune cells, such as CXCL5, and the lack of suitable chemokine receptors on T cells, decrease migration of CAR-T cells into tumors (Figure 1A) 24, 25.

Regional delivery of T cell through intraperitoneal and intra-tumoral injection is also a direct way to deliver CAR-T cells. In a Phase I clinical trial study, ErbB-targeting CAR-T cells were delivered via intra-tumoral injection to patients with head and neck squamous cell carcinoma 61. Local delivery is also suitable for other cancers, such as ovarian cancer and malignant pleural mesothelioma via the peritoneal and pleural cavities 22. However, innovative deliveries are needed to be developed for those patients with tumors that are not easy to access by regional delivery.

Stroma is mainly composed of ECM whose primary ECM component is heparan sulfate proteoglycan (HSPG) 22. Degradation of HSPG is the first step for T cells to pass stroma-rich solid tumors. Heparanase (HPSE) is an enzyme that degrades HSPG. Reduction of HPSE mRNA expression has been found in *in vitro-*expanded CAR-T cells, thus restricting their anti-tumor function in solid tumors due to the abundance of stroma 62. Caruanu, et al. had engineered CAR-T cells that express HPSE to promote T cell infiltration and antitumor activity. Their study showed improved capability of CAR-T cells to degrade the EMC 62. Wang et al. demonstrated that fibroblast activation protein (FAP)-targeted CAR-T cells enhanced cytotoxic function by decreasing tumor fibroblasts in animal models 27. VEGF receptor-2, highly expressed on tumor-associated endothelial cells, has been selected as another candidate for facilitating T cell infiltration 26. CAR-T cells engineered with VEGF receptor-2 generated a durable and increased tumor infiltration and enhanced antitumor effect (Figure 1A) 63. With anti-angiogenic pharmacologic intervention, tumor infiltration of CD8+ T cell is increased by blocking VEGF receptor-2 to achieve long-term therapeutic efficacy 64. It is clear that the complexity of genetic modification and long-term period of *in vitro* culture may also limit the clinical application of CAR-T cell therapy.

#### 6.2 Reversal of the immunosuppressive microenvironment

Preclinical data have shown that incorporation of costimulatory molecules into CARs helps CAR-T cells to reverse the immunosuppressive tumor microenvironment, for example, CD28 co-stimulation overcomes TGF-β-mediated repression of proliferation and enhances T-cell resistance to Treg cells 31, 32, 65. Burga et al. showed that MDSCs are responsible for liver metastases and inhibition of CEA-targeted CAR-T cells. Following MDSC depletion in a mouse model, the antitumor activity of CAR-T cells was rescued 33. During MDSC recruitment, tumor cells secrete high levels of granulocyte-macrophage colony-stimulating factor (GM-CSF) *in vivo.* Thus, GM-CSF neutralization might be an alternative method to inhibit MDSC expansion (Figure 1) 66, 67.

Inhibition of immunosuppressive cytokines by introducing a dominant-negative TGF receptor on CAR-T cells also improves the efficacy of CAR-T cells 68. In the tumor microenvironment, cytokine (e.g., IL-2, IL-12, and IL-15) activation could antagonize the effects of immunosuppressive factors and improve CAR-T cell efficacy. Studies have shown that the antitumor function is enhanced by CAR-T cells that co-express IL-12 (Figure 1B) 35, 69. Equally, IL-12 secretion by CAR-T cells has been shown to destroy antigen-negative cancer cells that may escape from the therapy 36. Other studies have confirmed that the antitumor effects of CAR-T cells are enhanced by IL-2 and IL-15 production 70-74. To rebalance the tumor microenvironment, armored CAR-T cells or redirected T cells for universal cytokine killing (TRUCKs) have been studied in preclinical trials. Koneru M et al. demonstrated that these armored CARs and TRUCKs secreted proinflammatory cytokines that induced transformation of the tumor microenvironment in mice with human ovarian cancer xenografts 75.

For treatment of cancers such as melanoma and renal cancer, the application of checkpoint inhibitors, such as anti-PD1, anti-CTLA-4 and anti-PD-L1, improves T cell responses in patients 41, 76. Preclinical data showed that blocking PD1-mediated immunosuppression also boosts the therapeutic effects of CAR-T cells (Figure 1B) 41. In a study of CAR-T cells with PD-1 blockade in a mouse model, Moon EK et al. found that PD-1 blockade improved the antitumor activity of human mesothelin-targeting CAR-T cells (Figure 1B) 77. HER2-targeted CAR-T cells in combination with anti-PD-1 significantly eliminated tumor cells in a mouse model 41. Suarez ER et al. engineered CAR-T cells to secrete anti-PD-L1 antibodies instead of administering anti-PD-L1 antibody 78. This approach not only reduced tumor progression but also enabled human NK cells to migrate to the tumor sites in a mouse model of renal carcinoma. NK cells exert the anti-tumor efficiency through antibody-dependent cell-mediated cytotoxicity (ADCC) and IFNγ stimulation of CD8^+^ T cells 22. Therefore, CAR-T cell therapy for solid tumors can be improved by infiltration of other immune cell subsets into the tumor microenvironment through local anti-PD-L1 antibody secretion. Interestingly, the number of MDSCs was also significantly diminished in the mouse tumor microenvironment. In addition, certain molecules, such as IL-6, may play double-sided roles in tumor microenvironment 79.

#### 6.3 Multiplexing CAR-T cells to target tumor profiles

Given by tumor heterogeneity and antigen escape variants, the next development in CAR-T cell therapy is to target more than one antigens, similar to the combinatorial strategy of traditional chemotherapy 80. This approach increases the chances of eliminating multiple sub-clonal populations simultaneously by targeting multiple TAAs or other factors in the tumor microenvironment.

There are various ways to create multi-specific CAR-T cells. The basic approach is to construct a pool with two unispecific CAR-T cell products, namely, a 'CAR pool', for simultaneous co-administration (Figure 1C) 81. A strategy of using combination targeting of HER2 and IL13R*α*2 by bispecific T cells in glioblastoma was shown to be more efficient in eliminating tumor cells and showed less antigen escape variants compared with the CAR-T cells targeting HER2 alone in both *in vitro* and *in vivo* mouse xenograft models 82. When treating lung cancer, a similar approach was applied to pool EphA2-targeted CAR and FAP*α-*targeted CAR to target the tumor microenvironment. This combination showed significant tumor killing *in vitro* and prolonged the survival of mouse xenografts compared with application of either CAR alone 83.

A single T cell platform can also possess dual antigen targeting when two (bispecific [bi]CARs)^83^ or more (triCARs) 84 unispecific CARs are expressed in T cells (Figure 1C) 81. In breast cancer, the proliferation of biCAR-T cells targeting HER2 and MUC1 *in vitro* was dependent on contact with both antigens simultaneously. biCAR-T cells coexpressing HER2 and IL13R*α*2 CAR molecules showed significant potential of eliminating tumor cells compared with unispecific CARs alone or pooled products in a glioblastoma model and the antigen escape variants also decreased substantially 83. In a non-small cell lung cancer model, the combination of PSCA- and MUC1-targeting CAR-T cells synergistically eliminated PSCA and MUC1 positive tumor cells 84. However, another study reported the biCAR-T cells did not enhance the level of IL-2, a marker of T cell activation compared with single CAR-T cells. This is possibly caused by the steric hindrance during simultaneously binding both antigens 85.

CAR-T function can be improved by alteration of the CAR configuration. In a proof-of-concept study, a tandem (tan) CAR was designed by Grada et al. that had two different scFv domains in tandem linked by a spacer (Figure 1C) 81. The tanCAR individually recognized each target antigen, and its function was strengthened when both antigens interacted with their scFv domain simultaneously *in vitro* and *in vivo* 85*.* This phenomenon suggests co-docking occurring to both target molecules. This concept was also established in an inducible *in vitro* targeting model and *in vitro* and *in vivo* antigen escape models 86. In addition to biCARs, triCARs, and tanCARs, neoantigen-like tissue factors (TFs) become promising novel antigens. In certain types of lung cancer, melanoma and other cancers, TFs are overexpressed on the surface of tumor cells, and TF-CAR-T cells were significantly activated to show specific cytotoxicity to TF-positive tumor cells* in vitro* 87.

Multispecific CAR-T cells have a potential advantage over unispecific CAR-T cells in avoiding on-target/off-tumor toxicities 49. In lymphoreticular malignancies, instead of CRS, multispecific CAR-T cells led to specific tissue cholangitis 88. A conditional biCAR was developed to minimize tissue damage (Figure 1C) 81. Different endomembrane signaling domains are included in the two CARs of T cells, and T cell activation depends on simultaneous interaction of both CARs with their specific TAAs. For example, in prostate cancer, a biCAR was constructed with two prostate cancer antigens (prostate-specific membrane antigen and prostate stem cell antigen), the expression of which are also found on normal tissues. There is a CD3ζ intracellular domain and a CD28-4-1BB domain that constitute a costimulatory intracellular domain. This special biCAR effectively targeted tissues expressing both antigens but not tissues positive with single antigen 89. Another example is that a conditional biCAR of mesothelin and *α*-folate receptor (FR*α*) with intracellular signaling domains of separated CD3*ζ* and CD28 are fully activated only when both antigens are present simultaneously in ovarian cancer. When multi-specific CAR-T cells were activated, cytokines were released and the tumor burden was reduced in both *in vitro* and* in vivo* experiments, similar to the single second generation of mesothelin-targeting CAR-T cells 90. An approach of adding a further layer of titratable control to activate the CAR-T cells was recently described by Wu et al. The structure contained a small molecule that forms a heterodimerized biCAR, consisting of a binding domain for the antigen and a domain for intracellular signaling (Figure 1C) 81, 91.

Multitargeting CARs have several potential advantages over conventional unispecific CAR molecules. Through cytokine release and cytolysis *in vitro* and decreasing the tumor burden *in vivo*, the antitumor effects of CAR-T cells may be further enhanced. Other structures, such as the tanCAR, may strengthen functionality by changing steric interactions with tumor antigens 81. However, the percentage of targeted tumor cells within tumor tissues should be concerned with multitargeting CARs 89, 92. There are still growing demands to find novel antigens as potent targets.

#### 6.4 Minimizing CAR-T cell toxicity

As mentioned above, to date, EGFRvIII is exclusively the tumor-specific CAR antigen, and it is fully restricted to human cancers and mostly found in glioblastoma 54, 55. EGFRvIII-positive tumor cells can be precisely targeted by specific CAR-T cells to enhance therapeutic efficacy and decrease the corresponding toxicity. In an animal model study of glioblastoma, EGFRvIII CAR treatment produced very promising results, and clinical trials are underway to test it on glioblastoma patients 93, 94.

Various targeting strategies have been developed to increase the specificity and safety of CAR-T cell therapy (Figure 1D) 27. One strategy is to modify T cells with two different CARs, and thus, tumor cells and normal cells can be induced to differentiate effectively. The first CAR molecule consists of the CD3ζ signaling domain, and the second CAR molecule contains the CD28 or CD137 costimulation signaling domain 27, 89, 90. Only when two CARs expressed in the same cells, the CAR-T cells can be fully activated. The on-target/off-tumor toxicities caused by TAA expression on normal tissues are limited by the CAR costimulatory domain on the same T cells. Preclinical data demonstrated that targeting multiple TAAs helps to minimize the possibility of antigen escape variant and efficiently target tumor subclones 81.

Furthermore, tumor specificity can be enhanced by an affinity-tuned CAR. In recent years, studies have demonstrated that adjusting the CAR affinity of CAR-T cells and maintaining potent antitumor efficacy *in vivo*, could distinguish tumor cells from normal cells with the expression of the same antigens at lower levels 51, 95. Thus, tuning CAR sensitivity through high scFv affinity in CAR-T cell therapy for overexpressed antigens in solid tumors provides a promising approach. In addition, an alternative strategy called inhibitory CAR (iCAR) can reduce unwanted off-target effects. iCARs recognize specific antigens that are expressed only on normal cells, thus protects CAR-T cell from attacking normal cells by induction of the negative signaling. Fedorov et al. demonstrated that an iCAR possessing PD-1 and CTLA-4 inhibition ability prevented normal tissues from off-target effects in mouse models, applying the principle of checkpoint inhibition to an antigen in the normal tissue but not in the tumor 96. However, due to the risk of iCAR to completely abolish T cell function, updated modifications, such as suicide genes, may be useful to control the unwanted toxicity 97.

The introduction of suicide switches, such as herpes simplex thymidine kinase (HSV-TK), inducible caspase 9 (iCasp9) and CD20, has already been clinically tested to control CAR-T cells 53, 98, 99. This smart “tumor sensing” strategy, with the balance of two-antigen recognition, potentially diminishes on-target/off-tumor toxicity 100. However, one disadvantage is the unintended elimination of the modified T cells. Transient RNA expression of CAR offers temporary redirected T cell activity and limits toxic side effects, even though, in solid tumor models, RNA CAR-T cell activity is limited by insufficient tumor infiltration 22.

## Companion diagnostics for CAR-T cells

Many reviews have exclusively discussed strategies to improve the efficacy of CAR-T cell therapy against solid tumors, while less attention has been paid to companion diagnostics and related detections. These diagnostics and detections are now becoming indispensable for CAR-T cell therapies as they are playing important roles in patient selection, disease outcome prediction, treatment regimen decisions, efficacy evaluation, and relapse prevention. In these regards, we would like to focus on the topics of detection of target antigen expression and the properties of CARs.

Since the death report of a metastatic colon cancer patient caused by on-target/off-tumor effects of ERBB2-targeted CAR-T cell therapy in 2010, the side effects of CAR-T cell therapy for solid tumors have received extensive attention 22, 43. The on-target/off-tumor effects of CAR-T cells are mainly caused by T cells attacking normal cells due to their expression of target antigens 50, 100. Thus, there is the compelling need for the identification of TSAs for treating solid tumors with CAR-T cells. Unless the depletion of those normal cells is tolerable, similar to CD19^+^ B cells, CARs with moderate affinity are considered to improve the effect of CAR-T cell therapy 2, 27, 46, 101, 102. Despite the lack of an ideal TSA, scientists have attempted to reduce the side effects of CAR-T cell therapies by investigating the extracellular domain of CARs, especially the affinity of the scFv domains. Studies have reported that reducing the affinity of CARs could spare normal cells that express the target antigen at a low level, thus avoiding the on-target/off-tumor effect 51, 95, 103. With detail comparisons between the T cell receptors and CARs, more precise adjustments have been made to CARs 104, 105. A CD38-targeted CAR with ~1,000-fold reduced affinity was confirmed to be more suitable for treating multiple myeloma, and an ICAM-1-targeting CAR with micromolar affinity was more appropriate than nanomolar counterparts for treating solid tumors 102, 106. In addition, the density of CARs on T cells is another factor that should be considered along with the affinity. Altogether, CARs with moderate affinity are suitable for targeting TAAs in solid tumors. However, there is still an important factor that impacts the affinity choice of CARs and should be considered: the density or expression level of the target antigens on tumor cells 107, 108.

To test the expression level of the target antigens on tumor cells, immunohistochemical (IHC) staining is ubiquitously utilized for regimen decisions. Another point that should be highlighted is the differences between the antibodies used for IHC and those for CARs, within which the scFv domains are responsible for binding the target antigens. Thus, using an antibody with the same clone origin for IHC and scFv for CARs has been emphasized 108, 109. Nevertheless, peptides may form different spatial structures under different circumstances, and this may hide some of the essential binding sites of the scFv domains compared with the related antibodies for IHC 110-113. Thus, the optimal antibody for IHC tests should bind the same epitopes as the relative CAR, or be in form of custom-made scFv peptide by coupling with a tag for secondary antibody detection.

**Figure 2:**
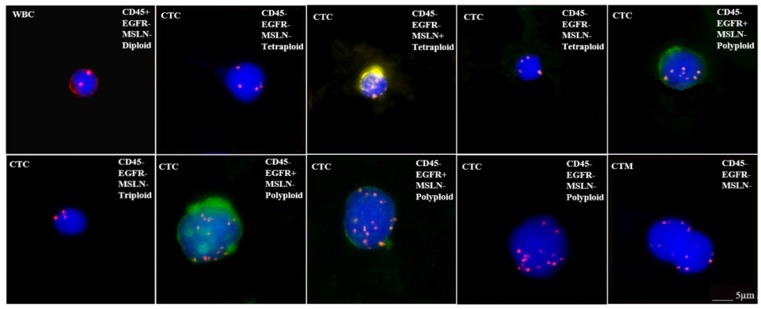
** Microscope images showing CTC analysis results in a bladder cancer patient.** CTCsare characterized as two mesothelin (MSLN)^+^ CTCs (expressing yellow), three EGFR^+^ CTCs (expressing green), MSLN^-^/EGFR^-^ CTCs and circulating tumor microemboli (CTM). In addition, each CTC is identified as triploid, tetraploid or polyploid by a FISH probe for CEP8. **WBC:** white blood cell; **CTC:** circulating tumor cell; **MSLN:** mesothelin; **CTM:** circulating tumor microemboli; **EGFR:** epidermal growth factor receptor

When discussing the detection of target antigen expression on tumor tissues using IHC assays, a question should be raised about the patients who do not have tumor tissue samples available such as in the situation of early stage of disease or metastasis, and minimal residual disease (MRD). In order to test whether those patients are suitable for respective CAR-T treatement, an alternative approach may be found by detecting the expression of target antigens on circulating tumor cells (CTC). We have tested the applicability of this protocol by examining the expression of EGFR and mesothelin on CTCs for CAR-T cell therapy, because these antigens are frequently targeted in clinical trials (Figure 2) 114. Our preliminary results as shown in Figure 2 demonstrated the feasibility for adapting CTC as one of the companion diagnostic and monitoring tool for CAR-T therapy. However, there are key factors that should be considered with this approach before it can become a reliable clinical testing utility. First, the number of CTCs varies with cancer types and stages. In order to assure sufficient CTCs are obtained and analyzed, the optimal sample blood volume still remain to be determined because too few CTCs may lead to a false negative result. Second, the epithelial-mesenchymal phenotype of CTCs must be considered because the epithelial-mesenchymal transition (EMT) process may hide the expression of target antigens 115, 116. Many currently available methods for CTCs enrichment do not take this into account, thus lead to another possibility for negative results. Finally, the correlation between the numbers of target antigen-positive CTCs and the therapeutic efficacy needs further investigation after treatment (Table 2). Beside IHC and CTC detection, many other methods and technologies, such as circulating tumor DNAs, circulating miRNAs, T-cell receptor sequencing/profiling and tumor mutation burdens, are currently being developed for companion diagnostics and efficacy monitoring for CAR-T cell therapy.

## Conclusions

In recent years, strategies have been applied to improve the efficacy and safety of CAR-T cell treatments for solid tumors, mainly through overcoming obstacles caused by the characteristics of T cells and tumor environment. Companion diagnostics, including IHC and CTC detection assays, can be applied to ameliorate the treatment of solid tumors with CAR-T cells.

## Figures and Tables

**Table 1:** Clinical trials in treatment of solid tumors in China by CAR-T cells

Antigen	Tumor target	Sponsor	Phase	NCT number	Study start
CD133	Liver cancer; pancreatic cancer; brain tumor; breast cancer; ovarian tumor; colorectal cancer, acute myeloid lymphoid leukemias	Chinese PLA General Hospital	Phase 1	NCT02541370	2015
CD138	Multiple myeloma	Chinese PLA General Hospital	Phase 1/Phase 2	NCT01886976	2013
CD19	Recurrent or refractory B-cell tumor	Second Military Medical University	Phase 1/Phase 2	NCT02644655	2015
CEA	Lung cancer; colorectal cancer; gastriccancer; breast cancer; pancreatic cancer	Southwest Hospital	Phase 1	NCT02349724	2014
Claudin 18.2	Advanced gastric adenocarcinoma, pancreatic adenocarcinoma	Changhai Hospital	NA	NCT03159819	2017
EGFR	Advanced EGFR-positive solid tumors	Chinese PLA General Hospital	Phase 1/Phase 2	NCT01869166	2013
EGFR	Advanced glioma	RenJi Hospital	Phase 1	NCT02331693	2014
EGFR	Advanced solid tumor	Shanghai Cell Therapy Research Institute	Phase 1/Phase 2	NCT03182816	2017
EGFR	EGFR-positive colorectal cancer	Shenzheng Second People's Hospital	Phase 1/Phase 2	NCT03152435	2017
EGFRvIII	Recurrent glioblastoma multiforme	Beijing Sanbo Brain Hospital	Phase 1	NCT02844062	2016
EGFRvIII	Glioblastoma multiforme	Shenzhen Geno-Immune Medical Institute	Phase 1/Phase 2	NCT03170141	2017
EphA2	EphA2-positive malignant glioma	Fuda Cancer Hospital	Phase 1/Phase 2	NCT02575261	2015
EpCAM	Liver neoplasms	Sinobioway Cell Therapy Co., Ltd.	NA	NCT02729493	2015
EpCAM	Stomach neoplasms	Sinobioway Cell Therapy Co., Ltd.	NA	NCT02725125	2015
EpCAM	Malignant neoplasms of the nasopharynx, TNM staging, distant metastasis and breast cancer recurrence	Sichuan University	Phase 1	NCT02915445	2016
EpCAM	Colon cancer, esophageal carcinoma, pancreatic cancer, prostate cancer, gastric cancer, hepatic carcinoma	First Affiliated Hospital of Chengdu Medical College	Phase 1/Phase 2	NCT03013712	2017
GD2	Neuroblastoma	Zhujiang Hospital	Phase 2	NCT02765243	2016
GD2	Relapsed or refractory neuroblastoma	Sinobioway Cell Therapy Co., Ltd.	NA	NCT02919046	2016
GD2	Solid tumor	Shenzhen Geno-Immune Medical Institute	Phase 1/Phase 2	NCT02992210	2016
GPC3	Hepatocellular carcinoma	RenJi Hospital	Phase 1	NCT02395250	2015
GPC3	Hepatocellular carcinoma and liver metastases	Shanghai GeneChem Co., Ltd.	Phase 1/Phase 2	NCT02715362	2016
GPC3	GPC3-positive hepatocellular carcinoma	Funda Cancer Hospital, Guangzhou	Phase 1/Phase 2	NCT02723942	2015
GPC3	Lung squamous cell carcinoma	Carsgen Therapeutics, Ltd.	Phase 1	NCT02876978	2016
GPC3	Hepatocellular carcinoma	Shanghai GeneChem Co., Ltd.	Phase 1/Phase 2	NCT03130712	2017
GPC3	Advanced hepatocellular carcinoma	Xinqiao Hospital of Chongqing	Phase 1/Phase 2	NCT03084380	2017
GPC3	Hepatocellular carcinoma, squamous cell lung cancer	Second Affiliated Hospital of Chengdu Medical University	Phase 1	NCT03198546	2017
GPC3	Hepatocellular carcinoma	RenJi Hospital	-	NCT03146234	2017
GPC3/MSLN/ CEA	Hepatocellular, pancreatic cancer, colorectal cancer	Shanghai GeneChem Co., Ltd.	Phase 1/Phase 2	NCT02959151	2016
HER2	Tumors refractory to chemotherapy and/or HER2 antibody therapy, advanced HER2-positive solid tumors	Chinese PLA General Hospital	Phase 1/Phase 2	NCT01935843	2013
HER2	Breast cancer, ovarian cancer, lung cancer, gastric cancer, colorectal cancer, glioma, pancreatic caner	Southwest Hospital	Phase 1/Phase 2	NCT02713984	2016
HER2	Breast cancer	Fudan Cancer Hospital	Phase 1/Phase 2	NCT02547961	2015
HerinCAR-PD1	Advanced malignancies	Ningbo Cancer Hospital	Phase 1/Phase 2	NCT02873390	2016
HerinCAR-PD1	Advanced solid tumors (lung, liver, and stomach)	Shanghai International Medical Center	Phase 1/Phase 2	NCT02862028	2016
MSLN	Malignant mesothelioma, pancreatic cancer; ovarian tumor; triple negative breast cancer; endometrial cancer; other mesothelin-positive tumors	Chinese PLA General Hospital	Phase 1	NCT02580747	2015
MSLN	Pancreatic cancer	Shanghai GeneChem Co., Ltd.	Phase 1	NCT02706782	2016
MSLN	Mesothelin-positive tumors	China Meitan General Hospital	Phase 1	NCT02930993	2016
MSLN	Solid tumors, adult advanced cancer	Ningbo Cancer Hospital	Phase 1/Phase 2	NCT03030001	2017
MSLN	Advanced solid tumors	Shanghai Cell Therapy Research Institute	Phase 1/Phase 2	NCT03182803	2017
MG7	Liver metastases	Xijing Hospital	Phase 1/Phase 2	NCT02862704	2016
MUC1	Malignant glioma of the brain; colorectal carcinoma; gastric carcinoma	PersonGen Biomedicine (Suzhou) Co., Ltd.	Phase 1/Phase 2	NCT02617134	2015
MUC1	Advanced refractory solid tumors (hepatocellular carcinoma, NSCLC, pancreatic carcinoma, triple-negative invasive breast carcinoma)	PersonGen Biomedicine (Suzhou) Co., Ltd.	Phase 1/Phase 2	NCT02587689	2015
MUC1	Advanced solid tumors	Shanghai Cell Therapy Research Institute	Phase 1/Phase 2	NCT03179007	2017
NY-ESO-1	Advanced NSCLC	Guangzhou Institute of Respiratory Disease	Phase 1	NCT03029273	2017
LMP1	Nasopharyngeal neoplasms	The Second Hospital of Nanjing Medical University	Phase 1/Phase 2	NCT02980315	2016
PD-L1 CSR	Glioblastoma multiforme	Beijing Sanbo Brain Hospital	Phase 1	NCT02937844	2016
PSCA/MUC1/ PD-L1/CD80/86	Advanced lung or other cancers	Second Affiliated Hospital of Guangzhou Medical University	Phase 1	NCT03198052	2017
PSMA/FRα	Bladder cancer, urothelial carcinoma bladder	Shenzhen Geno-Immune Medical Institute	Phase 1/Phase 2	NCT03185468	2017
Zeushield	NSCLC	Second Xiangya Hospital of Central South University	Phase 1	NCT03060343	2016

*The clinical trials are collected from clinicaltrials.gov

**Table 2:** Summary of application of companion diagnostics

Diagnostic Assays	Intentions	Related Issues	Suggestions
Immunohistochemistry (IHC)	Detecting the expression of targeting antigens for CAR-T cells	Different binding epitopes with scFv in CAR	Apply the same clone origin of the antibody for IHC and scFv for CAR; Custom synthesis of the scFv peptide by coupling with the substrate for the second antibodies.
Affinity tests	Reduce the side effects of CAR-T cells for solid tumors	Too high will cause on-target/off-tumor effects; Too low will cause inefficacy in killing cancer cells.	Moderate affinity is proven to be a better choice; Balance the affinity and density of CAR on the T cells; Balance the affinity of CAR and density of the antigens on the tumor cells.
Circulating tumor cells (CTCs)	Provide alternative for patients without tumor tissue; Evaluate therapeutic efficacy of CAR-T cells; Real-time monitoring of antigen-positive tumor cell relapse.	CTCs are rare in blood; CTCs with a mesenchymal phenotype may hide the expression of antigens.	Confirm the correlation between the positivity of the antigen-repressing CTCs and tumor tissues; Extract more blood than the traditional volume; Investigate the correlation between the number of targeting antigen-positive CTCs and the therapeutic efficacy needs.
